# Development of the young spine questionnaire

**DOI:** 10.1186/1471-2474-14-185

**Published:** 2013-06-12

**Authors:** Henrik Hein Lauridsen, Lise Hestbaek

**Affiliations:** 1 Research Unit for Clinical Biomechanics, Institute of Sports Science and Clinical Biomechanics, University of Southern Denmark, Clinical Locomotion Network, Campusvej 55, DK-5230, Odense M, Denmark; 2 Nordic Institute of Chiropractic and Clinical Biomechanics, Clinical Locomotion Network, Forskerparken 10A, 5230, Odense M, Denmark

**Keywords:** Children, Adolescents, Questionnaire, Spine, Prevalence, Pain

## Abstract

**Background:**

Back pain in children is common and early onset of back pain has been shown to increase the risk of back pain significantly in adulthood. Consequently, preventive efforts must be targeted the young population but research relating to spinal problems in this age group is scarce. Focus has primarily been on the working age population, and therefore specific questionnaires to measure spinal pain and its consequences, specifically aimed at children and adolescents are absent. The purpose of this study was to develop a questionnaire for schoolchildren filling this gap.

**Methods:**

The Young Spine Questionnaire (YSQ) was developed in three phases – a conceptualisation, development and testing phase. The conceptualisation phase followed the Wilson and Cleary model and included questions regarding spinal prevalence estimates, pain frequency and intensity, activity restrictions, care seeking behaviour and influence of parental back trouble. Items from existing questionnaires and the “Revised Faces Pain Scale” (rFPS) were included during the development phase. The testing phase consisted of a mixed quantitative and qualitative iterative method carried out in two pilot tests using 4^th^ grade children and focusing on assessment of spinal area location and item validity.

**Results:**

The testing phase resulted in omission of the pain drawings and the questions and answer categories were simplified in several questions. Agreement between the questionnaire prevalence estimates and the interviews ranged between 83.7% (cervical pain today) and 97.9% (thoracic pain today). To improve the understanding of the spinal boundaries we added bony landmarks to the spinal drawings after pilot test I. This resulted in an improved sense of spinal boundary location in pilot test II. Correlations between the rFPS and the interview pain score ranged between 0.67 (cervical spine) and 0.79 (lumbar spine).

**Conclusions:**

The Young Spine Questionnaire contains questions that assess spinal pain and its consequences. The items have been tested for content understanding and agreement between questionnaire scores and interview findings among target respondents. These preliminary results suggest that the YSQ is feasible, has content validity and is a well understood questionnaire to be used in studies of children aged 9 to 11 years.

## Background

The focus on research in adult spinal problems has fostered a plethora of back-specific outcome questionnaires related to the adult working population [1]. This led to the recommendation of a core set of adult outcome measures for low back in 1998 [2] and for chronic pain conditions in 2005 [3]. However, during the past decade it has become well established that the prevalence of spinal pain in children is high [4,5] and that the prevalence is gradually increasing during adolescence to approximate adult levels at the age of 18 [5,6]. Despite of this there are very few, if any, published questionnaires which focus on spinal problems and its consequences in children and adolescents.

Perusing the paediatric spinal literature reveals large heterogeneity in the reported prevalence estimates, illustrated in a review by Jeffries *et al*. who found lifetime prevalence estimates for low back pain ranging from 7% to 50.8% [5]. Several factors could be possible causes of the observed variation in prevalence figures. Firstly, the method of data collection (i.e. interviews vs. questionnaires vs. a mix of interviews and questionnaires) [7-10] and the administration setting (i.e. school vs. at home) [11-13] varies between and within studies. Secondly, the grouping and delineation of spinal areas differs between studies and lastly, most studies use a long (1-year or lifetime) recall period which is subject to recall bias [14-16].

As early onset of back pain has been shown to carry a significant risk of reporting back pain in adult life, and as this risk increases with increasing numbers of days with back pain during childhood [17-19], it seems only logical that primary prevention should target the young population and not, as has been the focus so far, the working adult population. Furthermore, considering the very high prevalence rates of spinal pain in the population, it is important to enable investigations into what constitutes negligible and significant spinal pain, as this will enable researchers to focus primary prevention on the group of children at increased risk of developing chronic spinal pain. For that purpose, simple prevalence estimates should be supplemented by information about frequency, intensity, disability and consequences.

Due to the lack of published standardised questionnaires aimed at children and adolescents we endeavoured to develop such a spinal questionnaire. The objective of this study was to develop and test a questionnaire measuring the prevalence and frequency of spinal problems in addition to pain intensity, activity restrictions, care seeking behaviour and influence of parental back trouble for children and adolescents.

## Methods

The development of the Young Spine Questionnaire (YSQ) was divided into three phases – a conceptualisation phase, a development phase and a testing phase.

### Conceptualisation of the YSQ

**Figure 1:**
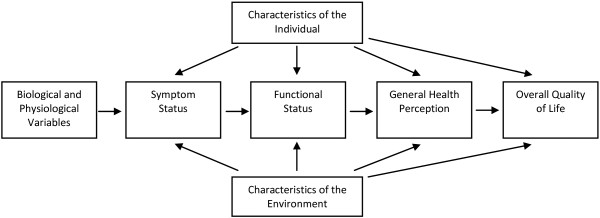
**The Wilson & Cleary conceptual model.***Note:* Adapted from Wilson et al. [21].

**Figure 2:**
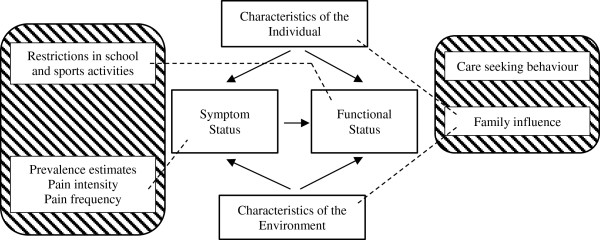
**Conceptual model of the young spine questionnaire.***Note:* The crossed-lined boxes represent the content of YSQ and the stippled lines indicate where the content belongs in the Wilson & Cleary conceptual model. The item on care seeking behaviour has no link as it did not fit the model.

The YSQ was designed as a self-report measure primarily to be used in cross-sectional cohort studies in early adolescence (9 to 11 years) [20]. It is based on the conceptual model of Wilson and Cleary (Figure 1) [21] and items were chosen to fit the symptom and functional status levels on the health continuum in addition to characteristics of the individual and environment (Figure 2). At the symptom status level, we included spinal prevalence estimates (daily, weekly and lifetime prevalence), frequency of pain episodes and pain intensity. At the functional status level, questions regarding restrictions in school and sports activities were chosen. Back pain in family members can relate to both characteristics of the individual and the environment. Parents’ *behaviour* during an episode of back pain has the potential to influence an individual psychologically (environment) whereas parents’ *opinions* about back pain may have an effect on symptom experience, personality and motivation, and finally there is a genetic component influencing the characteristics of the individual. For these reasons we linked family influence to both aspects of the model. Finally, an item mapping care seeking behaviour was included even though it did not fit the model. This was incorporated because it is considered an important consequence of back pain with the potential to be a proxy of severity.

Separate items are analysed on an individual basis and no summary scores are generated. Sections 1-3 are core questions of the YSQ whereas section 4 and 5 are optional and can be included or excluded according to the purpose of the study.

### Development of the YSQ

Initially, we constructed an item bank consisting of published questions and drawings fitting the conceptual model [10,18,22-25]. The back drawings were adapted from the Standardised Nordic Questionnaire [22] while the prevalence estimates questions came from a questionnaire used in a study of school children 8 to 16 years old [10,24,25]. Items regarding activity restrictions, care seeking behaviour and the influence of parental back trouble were adapted from a questionnaire used in the Funen Back Pain study [18].

To measure pain intensity we included a translated version of the “Revised Faces Pain Scale” (rFPS) which has been well validated in children aged 5 – 12 years [23]. We decided that the rFPS was the most suitable for several reasons. Firstly, the rFPS is a commonly used instrument in many trials (used in > 22 trials) which allows for comparing results [26]. Secondly, it is one of six well documented instruments in the targeted age group. In a review by Stinson *et al*. it was found to have good reliability, content validity, construct validity and responsiveness [27]. Thirdly, contrary to the original FPS, the revised version has enhanced scoring compatibility with other self-rating scales as it uses a common metric (0-5 or 0-10) [23,28]. Lastly, it has been shown that 85-90% of all children aged 9 or older reliably can provide self-report of pain given an age-appropriate scale under optimal conditions [29]. Permission to use the scale was obtained from the original developers.

As some of the items were developed for use with an adult population each item was carefully scrutinised and adapted to the reading capacity of children aged 9-12 in an expert group consisting of the authors and a professor in pedagogy and compulsory schooling. This iterative process resulted in the first draft version of the YSQ which consisted of 6 sections (YSQ-1).

Section 1-3 covered prevalence estimates and a pain scale relating to the severity of the cervical, thoracic and lumbar spine. At the beginning of each section a picture demarcating the location of the spinal area was included.

Section 4 included three questions measuring the consequences in terms of activity restrictions during sports, school absenteeism, and care seeking behaviour. Because self-reported pain is completely subjective, consequences of pain is often considered to be a proxy measure for severity of the pain, and as the consequences are more important than the pain itself from a societal point of view, we wanted to include this in the questionnaire. As reduced physical activity, seeking health care and staying at home a few days are the most common consequences reported in the literature [10,30], these became part of the questionnaire.

Section 5 covered the child’s perception of parental back trouble and its consequences. It is important to understand that this is not a measure of the parents’ actual spinal pain, but the children’s perception of it. This was included as several studies have reported a significant relationship between symptom reporting of family members and back pain [11,30-32].

Section 6 was two pain drawings (front and back) asking the child to locate areas of pain at other sites.

### Testing of the YSQ

**Figure 3:**
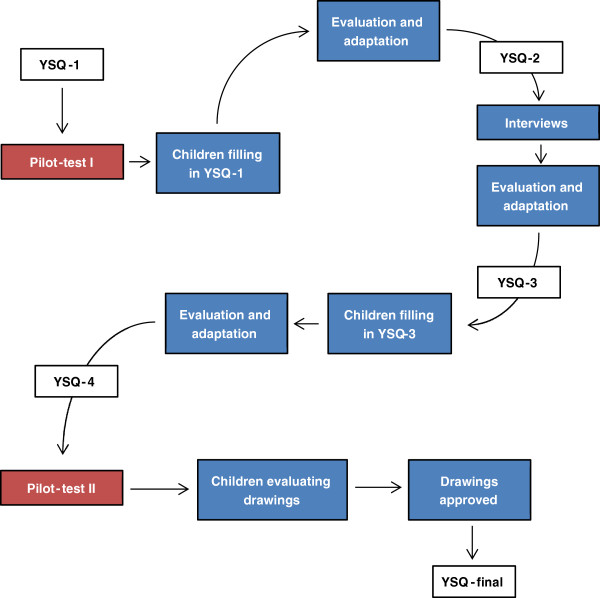
The testing and revision procedures of the young spine questionnaire.

We devised an iterative method to test and adapt the draft version of the YSQ for item understanding which consisted of two pilot tests (Figure 3). The method was a simplified version of the content validity method for assessing respondent understanding as proposed by Patrick et al. [33].

The study was not reported to the local ethics committee as this is not required according to the rules and regulations of the Danish scientific ethical committee [34].

#### Pilot test I

In pilot test I, the draft questionnaire items were tested on fourth grade high-school children 3 times during a normal school week. We recruited 52 children from 3 fourth grade high-school classes at a local school in Vissenbjerg, a Danish town of 3,000 inhabitants located at the centre of the island of Funen. Permission was obtained from the headmaster of the school and the class teachers. A detailed information and consent form was send to the parents of the participating classes containing the study purpose and detailing the procedures. If parents were unwilling to allow their child to participate they could contact the class teacher and the particular child would be excluded.

The children received the YSQ in the classroom during school hours at the beginning of the week without any instructions. During the test a member of the research team was present and the children were allowed to ask questions if they had problems answering any of the items. The post-test analysis screened questions which arose during the test in addition to missing or illogical answers found in the questionnaires. This analysis resulted in revision of several items of the questionnaire (YSQ-2).

Two days later the research team interviewed all children using a semi-structured interview template. The semi-structured questions were designed to obtain the same information as contained in all 6 sections of the YSQ, however, using different semantics and open-ended questions. For example, to test if the children understood the picture outlining the neck, the picture of the neck from the YSQ was shown and explained to each child. Following this, each child was asked to show the upper and lower boundaries of the neck on themselves. Similarly, we asked if they had or have had pain in the area in question. If they answered confirmatively, they were asked when it was and to rate the pain intensity on an 11-box numeric rating scale (NRS) [35], which could then be correlated with the rFPS. A post-interview analysis of all the interviews was conducted resulting in additional revision of the questionnaire (YSQ-3).

The YSQ-3 was administered to the children at the end of the week testing the feasibility and validity of the revisions. This time the children were not allowed to ask questions and the filled-in questionnaires were compared to the previous questionnaires and interview responses. This led to new revisions of the pain drawings and a few minor changes (YSQ-4).

#### Pilot test II

Since the drawings demarcating the spinal areas were changed in YSQ-4, we performed a second pilot test among 18 children from a fourth grade high-school class in the municipality of Horsens. Each child was isolated in a room and shown the new drawings one by one. They were asked to point out the demarcations of the three spinal areas on themselves or on the interviewer.

#### Cross-cultural translation

A cross-cultural translation of the rFPS (English version) into Danish was carried out according to the guidelines outlines on the official homepage [36]. An electronic version of the Danish rFPS can be found on “http://www.spoergeskemaer.dk/spoergeskemaer-om-smerter-hos-boern”. This translation was subsequently adapted to fit the structure of the YSQ. Furthermore, we completed a cross-cultural translation of the final version of the YSQ into English which followed stage I to III of the guidelines developed by Beaton et al. [37]. The full version of the questionnaire can be found in Additional file 1 (English) and Additional file 2 (Danish) and electronic versions can be found on “http://www.spoergeskemaer.dk/young-spine-questionnaire”.

### Statistical analyses

In the testing phase we calculated descriptive statistics for the children in pilot test I (YSQ-1) and II (YSQ-4). In addition, percentage agreement was used between the prevalence estimates from the questionnaire (YSQ-1) and the interviews for two reasons. Firstly, it highlighted problematic items which subsequently could be adapted. Secondly, it shows how well the question wording corresponded with the children’s own explanation of their problem. Finally, median, inter-quartile range and Spearman’s correlations were calculated between the 11-box numeric rating scale and the rFPS (YSQ-1).

## Results

### Participants

The children participating in pilot test I had a mean age of 10 with slightly less girls (47.2%) compared to boys (Table 1). Based on YSQ-1 a high proportion of the children reported that they had experienced low back pain (47.2%) or neck pain (41.5%) while slightly less had experienced thoracic pain (28.3%) at least once in their life. The 18 children participating in pilot test II also had a mean age of 10 and there were 11 girls (61%). The focus of pilot test II was on the pain drawings for which reason no spinal pain data is available.

**Table 1:** Descriptive statistics of the children in pilot test I (YSQ-1†)

**Characteristic**	**Fourth grade children (*n*** **= 53)**
Age, mean (min., max.)	10 (9-11)
Sex, female, %	47.2
Lifetime prevalence*, %	
*Cervical pain*	41.5
*Thoracic pain*	28.3
*Lumbar pain*	47.2
1-week prevalence, %	
*Cervical pain*	18.8
*Thoracic pain*	3.8
*Lumbar pain*	11.3
Point prevalence, %	
*Cervical pain*	9.4
*Thoracic pain*	3.8
*Lumbar pain*	0.0

* Question 1a, 2a and 3a dichotomised by grouping answer category “Often” and “Once in a while” to “Yes”.

† Results reported on the basis of Young Spine Questionnaire version 1.

### Testing of the YSQ

#### Pilot test I

During the interview the children were tested for agreement of the upper and lower boundaries of the cervical, thoracic and lumbar spine between the drawings included in the questionnaire (see Additional file 1) and the interview findings. Using the YSQ-1 the children were good at identifying the borders of the cervical spine (both boundaries correctly identified: 91.8%) but the lumbar (67.4%) and thoracic spines (63.3%) were somewhat less clearly recognised (Table 2). Consequently, we added several easily recognisable bony landmarks such as an outline of scapula, lines for the 12^th^ ribs and gluteal folds to aid the children in recognizing the regions of the spine properly. Furthermore, the interviews revealed that the children included pain from the coccyx (most often due to falls) as low back pain (YSQ-2). As a result we modified the adult drawings [22] to not include the gluteal areas and move the lower border of the lumbar spine up to midway on the sacrum.

**Table 2:** Understanding of location of the spinal regions based on interviews

**Region**	**Correctly identified (%)**	
	Pilot test I: YSQ-1* (*n* **= 49)**	Pilot test II: YSQ-4* (*n* **= 18)**
Cervical spine		
*Both boundaries*	91.8	83.3
*Upper boundary*	95.9	100
*Lower boundary*	95.9	83.3
*None*	0.0	0.0
Thoracic spine		
*Both boundaries*	63.3	94.4
*Upper boundary*	85.7	100
*Lower boundary*	71.4	94.4
*None*	6.1	0.0
Lumbar spine		
*Both boundaries*	67.4	72.2
*Upper boundary*	77.6	83.3
*Lower boundary*	81.6	83.3
*None*	8.2	5.5

*Young Spine Questionnaire version 1 and 4.

Agreement between the questionnaire prevalence estimates (YSQ-1) and the interviews was also tested (Table 3). This showed agreement estimates ranging between 83.7% (cervical pain today) to 97.9% (thoracic pain today). We also scrutinised the response options during the interviews. This resulted in changing the response categories of several questions as the children had difficulty understanding their meaning.

**Table 3:** Agreement between questionnaire (YSQ-1*) and interview of prevalence estimates

**Prevalence estimates**	**Agreement (%)**
Cervical spine pain (*n* = 49)	
*Ever*	87.8
*Last week*	85.7
*Today*	83.7
Thoracic spine (*n* = 49)	
*Ever*	81.6
*Last week*	93.9
*Today*	97.9
Lumbar spine (*n* = 49)	
*Ever*	89.8
*Last week*	85.7
*Today*	93.9

*Young Spine Questionnaire version 1.

The score of the rFPS (YSQ-1) was compared to the NRS score from the interviews (Table 4). Correlations ranged from 0.67 for the cervical spine to 0.79 in the lumbar spine.

**Table 4:** Median, interquartile range (IQR), and correlations between NRS and rFPS according to spinal level

	**Interview**			Questionnaire (YSQ-1*)			
**Spinal level**	**NRS (0-10)**			**rFPS (0-5)**			***r***_**(NRS, rFPS)**_
	***n***	**Median**	**IQR**	***n***	**Median**	**IQR**	
Cervical	49	3	4	53	2	2	0.67
Thoracic	49	0	3	53	0	1	0.76
Lumbar	49	0	3	53	0	1	0.79

*Young Spine Questionnaire version 1.

Lastly, section 5 (the child’s perception and consequences of parental back trouble) and section 6 (pain drawings of additional sites of pain) were changed fundamentally during pilot test 1 (YSQ-1). Due to many unanswered questions it was decided to split question 5 into 2 sections; one relating to the mother’s back pain and consequences thereof and another to the father’s. Section 6 asked the child to circle other areas of pain on a pain drawing. This gave rise to a variety of very different responses ranging from no other pain to pain almost everywhere. The interviews disclosed widespread misinterpretation of the pain drawing as many children included small injuries as scraping their knee during a break or a small knock to the arm during sports. Consequently, it was decided to completely remove section 6 from the questionnaire and the YSQ-3 and onwards only consisted of 5 sections.

#### Pilot test II

The drawings from YSQ-4 (pilot test I) were tested in pilot test II. The percentage of correctly identified boundaries for each region ranged from 72.2% - 100% (Table 2). Compared to pilot test I there was a significant improvement in the amount of children being able to demarcate the thoraco-lumbar junction (lower border of the thoracic spine and upper border of the lumbar spine) whereas the lower boundary of the neck was slightly less well delineated.

#### Cross-cultural translation

The rFPS was translated into Danish with no notable issues. Subsequently, it was adapted to fit the structure of the full questionnaire 1) by modifying the vignette to be patient/respondent driven and not interviewer driven and 2) by adding the verbal descriptors (anchors) from the vignette above the upper and lower end pictures.

The translation of the YSQ-final revealed only a few noteworthy issues. Firstly, the semantics for each question or answer category was chosen to match the ability of the age group. Consensus was reached by scrutinising common language and conceptual equivalence. Secondly, the translators discussed how to sensibly translate question 5a (father) and 5c (mother). The direct translation of question 5a reads: *“Has your father (him you live with) ever had back or neck pain?”.* The Danish wording was chosen as a small but important minority of children left the questions unanswered due to problems interpreting ‘father’ when living in a divorced family. Consensus was agreed on the wording *“Has your father or stepfather ever had neck or back pain?”*, as it was felt that the children would intuitively relate to the person with the largest influence on their behaviour. Similar considerations were made for the mother in question 5c.

## Discussion

The Young Spine Questionnaire is a much needed novel questionnaire measuring childhood and early adolescent spinal pain prevalence rates in addition to pain intensity, activity restrictions, care seeking behaviour and the possible influence of parental back trouble in a standardised fashion. Our preliminary results suggest that the YSQ is feasible, have valid spinal pain prevalence estimates and pain scores and is phrased in such a manner that the target population has an acceptable level of item understanding.

### Drawings

The drawings of the spinal areas included in the YSQ were adapted from the Standardised Nordic Questionnaires which originally were developed for the adult population [22]. Our results demonstrate that children at the age of 10 have a different understanding of the boundaries of especially the thoracic and lumbar spine compared to adults. Many found it difficult to localise the transition from the thoracic to the lumbar spine which might be due to immaturity of the children’s anatomical knowledge. Also, most adults have an understanding of the concept “low back pain” which most of the children in this age group have not yet developed. Changing the drawings to include more bony landmarks helped to avoid these problems.

The original drawings also included the buttocks and thus the coccyx. Therefore, the frequent falls (e.g. from play and sports) on the buttocks causing coccygeal pain and/or bruised buttocks would be classified as low back pain. In the original drawing, the buttocks are included to capture pain radiating from the lumbar spine to the buttocks, but due to a high rate of non-spinal buttock/coccyx pain in this age group, it was a necessary trade-off to exclude the buttocks from the drawing.

In all the test phases, the sequence of sections was the same (cervical, thoracic, lumbar). Since only very few of the children had problems identifying the neck area, this may have been used as a reference point improving the identification of the other areas. It is not known, how the drawings of the thoracic and lumbar areas will be perceived if they are shown in another sequence or used individually.

### Prevalence estimates

The YSQ contain a frequency question (which can be dichotomised to represent lifetime prevalence) as well as point and 1-week prevalence estimates for all the spinal regions. Pilot test I revealed prevalence estimates of 41.5% and 18.8% (lifetime and 1-week) for the cervical spine, 28.3% and 3.8% for the thoracic spine and 47.2% and 11.3% for the lumbar spine. These estimates agreed well with findings from the interviews which ranged between 85.7-97.9% and we therefore believe that the prevalence questions accurately reflect the children’s perception of pain prevalence. In addition, our estimates were similar to reported lifetime and 1-week estimates in the same age group which ranged from 18.8-51.0% [38] and 23-44.3% [31,39] in the cervical spine, 9.5-72% [40,41] and 3.4-51.4% [31,42] in the thoracic spine, and 7.0-72.0% [6,41] and 9.5-20.0% [39,43] in the lumbar spine. To our knowledge no point estimates have been reported in the literature for comparison. On the basis of this, we believe that by asking for specific answer categories and subsequently collapse them for analyses, a more precise estimate can be obtained compared to simply asking “Have you ever had pain?”. However, because the prevalence estimates are based on the first version of the questionnaire and due to the small sample sizes these estimates should be interpreted with caution.

When developing the YSQ we decided not to include a focused lifetime prevalence estimate question due to the risk of “memory decay” where children are more likely to forget ‘episodes’ of spinal problems with the passage of time [16]. Secondly, “forward telescoping of events”, a tendency to recollect episodes of e.g. spinal problems as having happened more recently, are more likely to bias estimates with long recall periods [15]. Instead we included a frequency of spinal problems question which does not focus on a set time-frame. If needed for comparison to other studies, this can be converted into lifetime prevalence by collapsing the three last answer categories. However, we believe there should be more focus on frequency and duration, as a single short period of back pain is less likely to have long term consequences than more long-lasting or recurrent back trouble [17].

### Pain ratings

For each of the three spinal regions we added the rFPS as measure of pain intensity. To our knowledge the rFPS has not been used in spinal pain studies of children or adolescents, and it is therefore unclear how well the pain scale performs for this condition. However, our results showed acceptable correlation between the rFPS and the NRS (*r* = [0.67-0.79]) supporting the validity of the rFPS also for spinal pain. Similar results were found in a study of post-operative pain ratings comparing the same scales (*r* = 0.89) [44].

In the Danish translation of the rFPS we used “rigtig meget ondt” (very much pain) rather than the commonly used adult version “værst tænkelige smerte” (worst pain imaginable) as the latter is considered difficult for children to understand since they have a limited experience of pain intensity [29].

### School, leisure, treatment and family

Section 4 of the questionnaire contains questions related to activity restrictions (physical activity and school absenteeism) and care seeking behaviour (treatment received). We included these questions as proxy measures for severity of pain, and in combination with the rFPS they may have the potential to distinguish trivial from significant pain. Our experience from the pilot-tests is that children’s reporting of pain is more spontaneous and immediate compared to adults resulting in a higher frequency of trivial pain being recorded when answering the prevalence questions. This supported our decision to include these measures, so it is possible to some degree to qualify the answers in order to target primary intervention programmes to children with high risk of developing spinal pain in their adult life.

The last section measures the child’s perception and consequences of the parents back trouble. These questions had to be completely rephrased to be very specific, illustrating the need for testing questions in the target population. We believe children to a certain extent will imitate their parents’ behaviour reinforcing the relevance of the information when interpreting the children’s answers to the participation questions in section 4.

After the completion of final version of the YSQ it was decided to include section 4 and 5 as optional since they may not be relevant in studies which primarily focus on prevalence.

### Strength and limitations

The study has several strengths. Firstly, the questionnaire includes all 3 spinal areas and considers each as a distinct region [10]. Secondly, the emphasis on relevant content and content understanding of the target population during the development phase resulted in high content validity of the questionnaire. This was achieved using an iterative process combining ordinary questionnaire data with interview data.

Several drawbacks need to be mentioned. First of all, the questionnaire needs to be tested for reliability. Secondly, the questionnaire has only been tested on school-children in the age range of 9-11 years and cannot be generalised to other age groups. Thirdly, the iterative process and testing of the YSQ was mainly performed on preliminary versions (YSQ-1 to YSQ-3). It resulted in changes to all included items (either the question or the answer categories) and drawings for which reason caution is advised when applying these results to the final version. We recommend that future validation studies include a thorough evaluation of the items in the final version of the YSQ. Finally, we reinforce that the questionnaire has been developed and tested for use in cross-sectional cohort studies. At this point in time it is not recommended to use it in a longitudinal setting measuring change for several reasons: 1) the questions and response options have not been designed for the purpose of being responsive to change, and 2) the questionnaire does not have a summary score and change scores are therefore reliant on the validity of scores on single questions. Future studies need to ascertain whether or not the included questions can be used in a longitudinal design, measuring change.

## Conclusions

The Young Spine Questionnaire is a novel self-report questionnaire designed to measure spinal pain prevalence, pain intensity, the consequences in terms of activity restrictions, school absenteeism and care seeking behaviour, and finally the influence of parental back trouble. Feasibility, item understanding and item agreement between questionnaire scores and interview findings were acceptable, and from our preliminary findings we conclude that the YSQ has content validity, is well understood by the target population and can be used in cross-sectional cohort studies of children aged 9 to 11 years. Further psychometric testing is warranted.

## Competing interests

The authors declare that they have no competing interests.

## Authors' contributions

HHL and LH generated the concept, study design and performed the quantitative and qualitative research. Both authors participated in the development of the manuscript and review. HHL performed the statistical analyses. All authors read and approved the final manuscript.

## Pre-publication history

The pre-publication history for this paper can be accessed here:

http://www.biomedcentral.com/1471-2474/14/185/prepub

## Supplementary Material

Additional file 1 English version of the ‘Young Spine Questionnaire’. Click here for file

Additional file 2 Danish version of the ‘Young Spine Questionnaire’. Click here for file
