# Supplementary material for: Development of the young spine questionnaire
Source: BMC Musculoskelet Disord. 2013 Jun 12;14:185. doi: 10.1186/1471-2474-14-185 (PMC3689606; doi:10.1186/1471-2474-14-185)
Supplement: Additional file 1 — English version of the ‘Young Spine Questionnaire’. [file 1471-2474-14-185-S1.docx]

## Appendix 1. English version of the ‘Young Spine Questionnaire’

| **Name______________________________ Class________________**  This questionnaire is related to the spine and neck. Use only one cross (X) to answer each question. If none of the answers are suitable, place your cross by the answer that is best suited. | | | | | | | | | |
| --- | --- | --- | --- | --- | --- | --- | --- | --- | --- |
|  | | | | | |  | | | |
| **1. The neck is shown in the picture** | | | | | |  | | | |
|  | | | | | |  | | | |
|  | | | 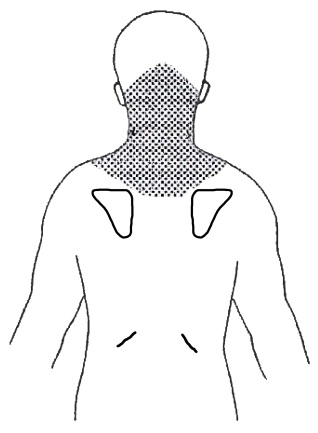  The neck | | | | |  | |
|  | | | Person seen from behind | | | | |  | |
|  | | |  | | | | |  | |
|  | | |  | | | | |  | |
| 1a. Have you had pain in the neck? | | | | | | 🞏 Often  🞏 Once in a while  🞏 Once or twice  🞏 Never | | | |
|  | | | | | |  |  |  |  |
|  | | | | | |  |  |  |  |
|  | | | | | |  | | | |
| 1b. Have you had neck pain in **the last week**? | | | | | | 🞏 Yes  🞏 No | | | |
|  | | | | | |  |  |  |  |
|  | | | | | |  | | | |
| 1c. Have you had neck pain **today**? | | | | | | 🞏 Yes  🞏 No | | | |
|  | | | | | |  |  |  |  |
|  | | | | | |  | | | |
| The faces below show how much something can hurt. The pain ranges from ‘No pain’ to ‘A lot of pain’. | | | | | | | | | |
|  | | | |  | | | | | |
| 1d. Put a cross (X) on the face which shows how much pain you have had in the neck when it was worst. | | | | | | | | | |
|  | | | | |  | | | | |
| No pain |  |  | | |  | |  | | A lot of pain |
| 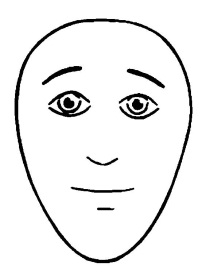 | 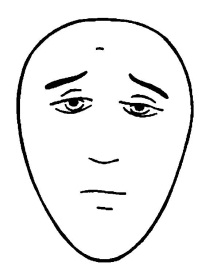 | 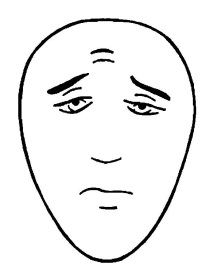 | | | 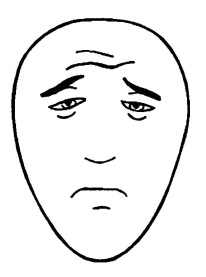 | | 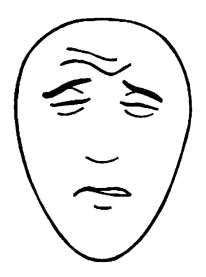 | | 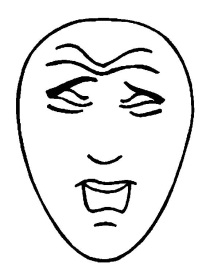 |

| **2. The middle of the back is shown in the picture.** | | | |  | | |
| --- | --- | --- | --- | --- | --- | --- |
|  | | | |  | | |
|  | | 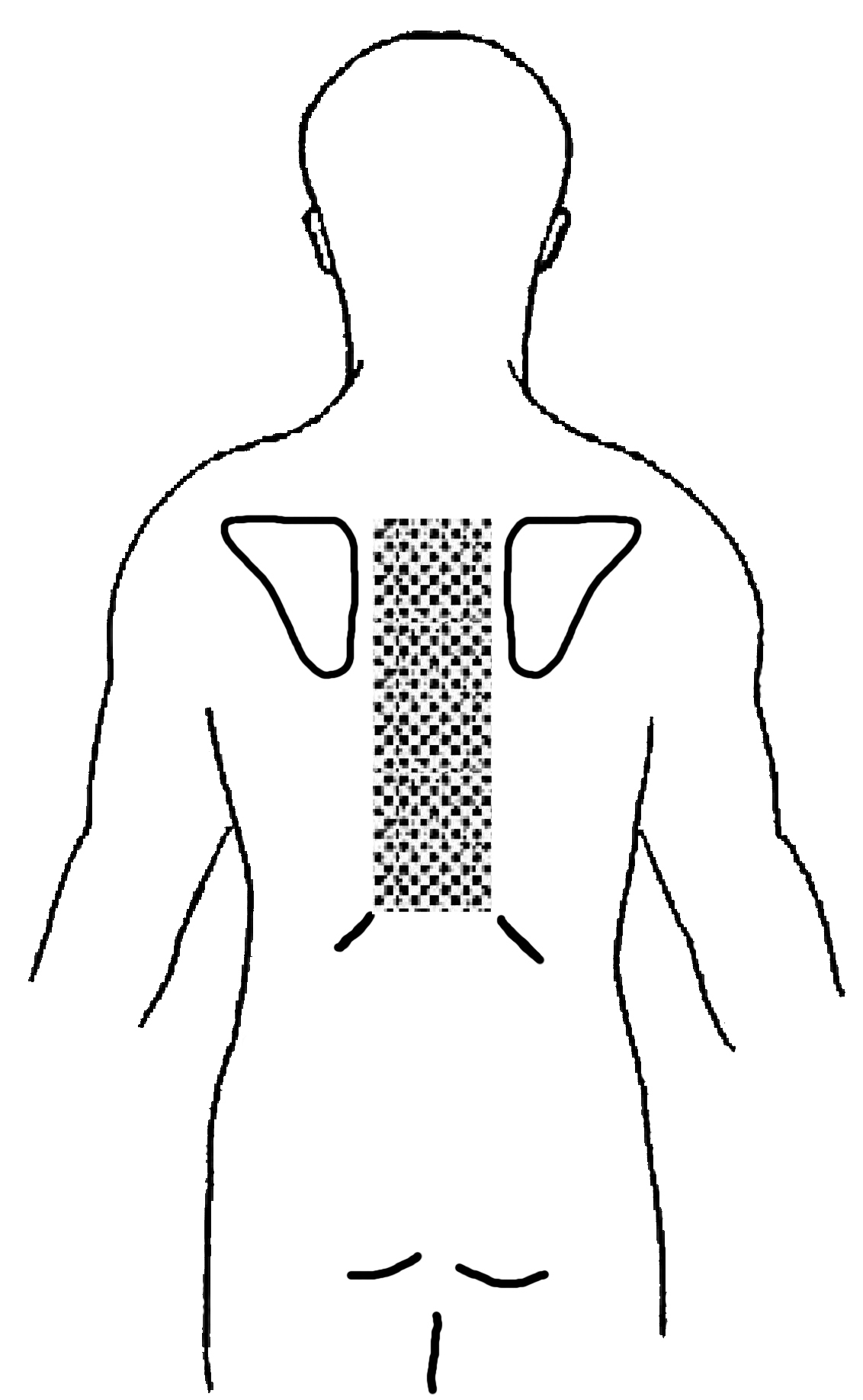  Middle of the back | | |  | |
|  | | Person seen from behind | | |  | |
|  | | | |  | | |
| 2a. Have you had pain in the middle of the back? | | | | 🞏 Often  🞏 Once in a while  🞏 Once or twice  🞏 Never | | |
|  | | | |  |  |  |
|  | | | |  |  |  |
|  | | | |  | | |
| 2b. Have you had pain in the middle of the back in **the last week**? | | | | 🞏 Yes  🞏 No | | |
|  | | | |  | | |
| 2c. Have you had pain in the middle of the back **today**? | | | | 🞏 Yes  🞏 No | | |
|  | | | |  |  |  |
|  | | | |  | | |
| 2d. Put a cross (X) on the face which shows how much pain you have had in the middle of the back when it was worst. | | | | | | |
|  | | | |  | | |
| No pain |  |  |  | |  | A lot of pain |
| 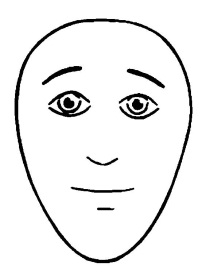 | 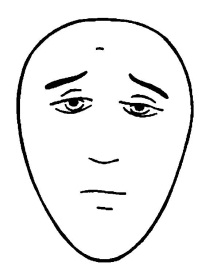 | 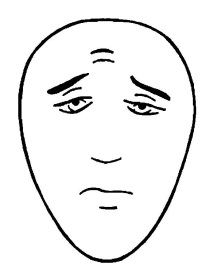 | 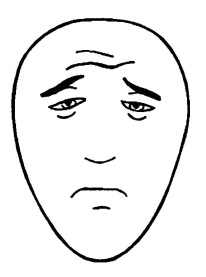 | | 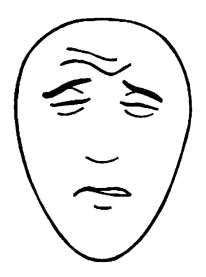 | 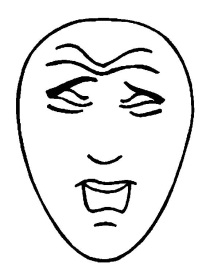 |

| **3. The lower back is shown in the picture.** | | | | |  | | | |
| --- | --- | --- | --- | --- | --- | --- | --- | --- |
|  | | | | |  | | | |
|  | | | 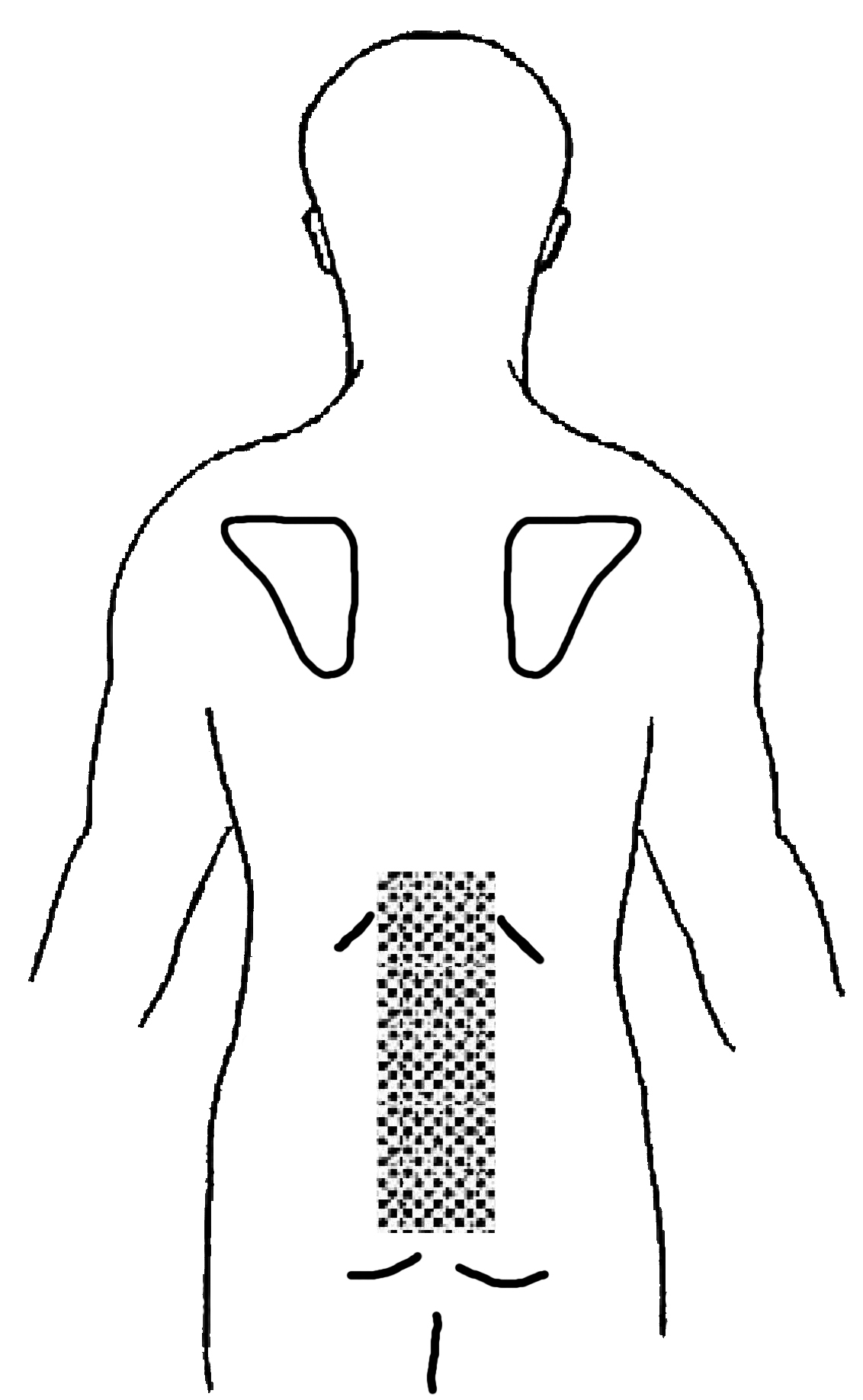  The lower back | | | |  | |
|  | | | Person seen from behind | | | |  | |
|  | | | | |  | | | |
| 3a. Have you had pain in the lower back? | | | | | 🞏 Often  🞏 Once in a while  🞏 Once or twice  🞏 Never | | | |
|  | | | | |  |  |  |  |
|  | | | | |  |  |  |  |
|  | | | | |  | | | |
| 3b. Have you had pain in the lower back in **the last week**? | | | | | 🞏 Yes  🞏 No | | | |
|  | | | | |  |  |  |  |
|  | | | | |  | | | |
| 3c. Have you had pain in the lower back **today**? | | | | | 🞏 Yes  🞏 No | | | |
|  | | | | |  |  |  |  |
|  | | | | | | | | |
| 3d. Put a cross (X) on the face which shows how much pain you have had in the lower back when it was worst. | | | | | | | | |
|  | | | |  | | | | |
| No pain |  |  | |  | |  | | A lot of pain |
| 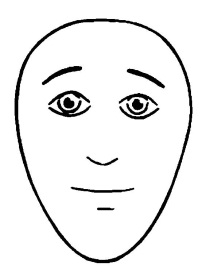 | 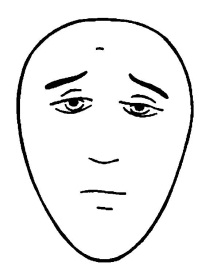 | 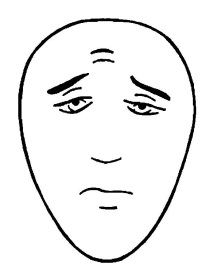 | | 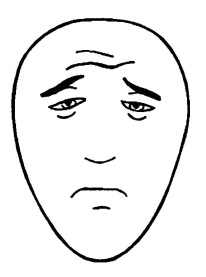 | | 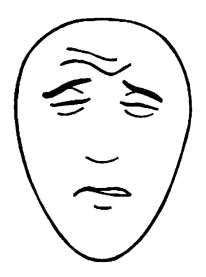 | | 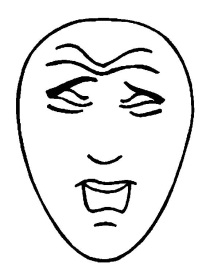 |

| **4. School, recreation and treatment** |  | |  |
| --- | --- | --- | --- |
|  |  | |  |
| 4a. Have you stayed home from school because of neck or back pain? | | 🞏 Often  🞏 Once in a while  🞏 Once or twice  🞏 Never | |
|  | |  |  |
|  | |  |  |
|  | |  | |
| 4b. Has neck or back pain sometimes stopped you from doing sports? | | 🞏 Often  🞏 Once in a while  🞏 Once or twice  🞏 Never | |
|  | |  | |
| 4c. Have you been to a doctor, chiropractor or physiotherapist because of neck or back pain? | | 🞏 Often  🞏 Once in a while  🞏 Once or twice  🞏 Never | |
|  | |  |  |
|  | |  |  |
| **5. The family** | |  |  |
|  | |  |  |
| 5a Has your **father** or stepfather ever had back or neck pain? | | 🞏 Yes  🞏 No |  |
|  | |  |  |
| 5b. If he has, has it kept him home from work? | | 🞏 Often  🞏 Once in a while  🞏 Never | |
|  | |  |  |
|  | |  |  |
| 5c Has your **mother** or stepmother ever had back or neck pain? | | 🞏 Yes  🞏 No |  |
|  | |  |  |
| 5d. If she has, has it kept her home from work? | | 🞏 Often  🞏 Once in a while  🞏 Never | |
|  | |  |  |
