# Supplementary material for: Development of the young spine questionnaire
Source: BMC Musculoskelet Disord. 2013 Jun 12;14:185. doi: 10.1186/1471-2474-14-185 (PMC3689606; doi:10.1186/1471-2474-14-185)
Supplement: Additional file 2 — Danish version of the ‘Young Spine Questionnaire’. [file 1471-2474-14-185-S2.docx]

## Appendix 2. Danish version of the ‘Young Spine Questionnaire’

| **Navn______________________________ Klasse________________**  Dette spørgeskema handler om ryggen og nakken. Du må kun sætte ét kryds ved hvert spørgsmål. Hvis ingen af svarene passer helt på dig, så sæt kryds ved det, der passer bedst. | | | | | | | | |
| --- | --- | --- | --- | --- | --- | --- | --- | --- |
|  | | | | |  | | | |
| **1. Nakken sidder som vist på billedet** | | | | |  | | | |
|  | | | | |  | | | |
|  | | | 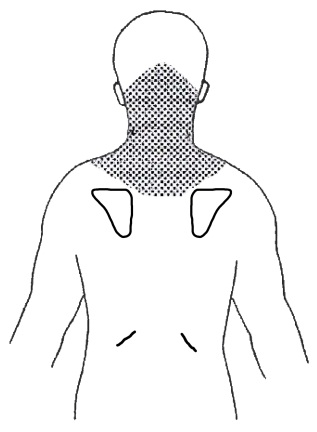  Nakken | | | |  | |
|  | | | Person set bagfra | | | |  | |
|  | | |  | | | |  | |
|  | | |  | | | |  | |
| 1a. Har du haft ondt i nakken? | | | | | 🞏 Ofte  🞏 En gang imellem  🞏 En eller to gange  🞏 Aldrig | | | |
|  | | | | |  |  |  |  |
|  | | | | |  |  |  |  |
|  | | | | |  | | | |
| 1b. Har du haft ondt i nakken **den sidste uge**? | | | | | 🞏 Ja  🞏 Nej | | | |
|  | | | | |  |  |  |  |
|  | | | | |  | | | |
| 1c. Har du ondt i nakken **i dag**? | | | | | 🞏 Ja  🞏 Nej | | | |
|  | | | | |  |  |  |  |
|  | | | | |  | | | |
| Ansigterne nedenfor viser hvor meget noget kan gøre ondt. Smerten stiger fra ”ikke ondt” til ”rigtig meget ondt”. | | | | | | | | |
| 1d. Sæt kryds over det ansigt, der viser hvor ondt du havde i nakken, da det var værst | | | | | | | | |
|  | | | |  | | | | |
| Ikke ondt |  |  | |  | |  | | Rigtig meget ondt |
| 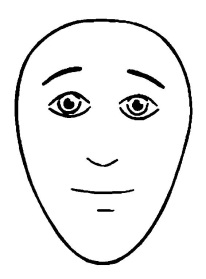 | 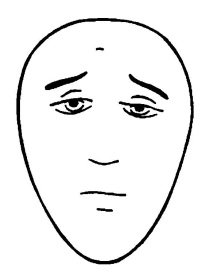 | 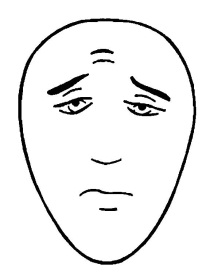 | | 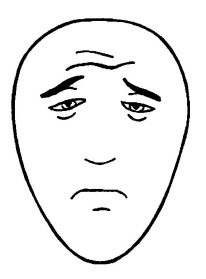 | | 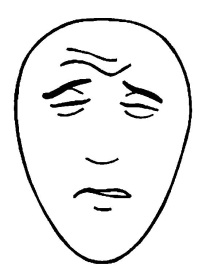 | | 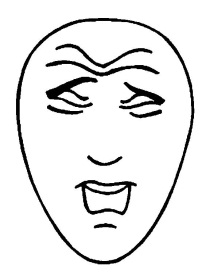 |

| **2. Midt på ryggen er som vist på billedet** | | | | |  | | | | |
| --- | --- | --- | --- | --- | --- | --- | --- | --- | --- |
|  | | | | |  | | | | |
|  | | | 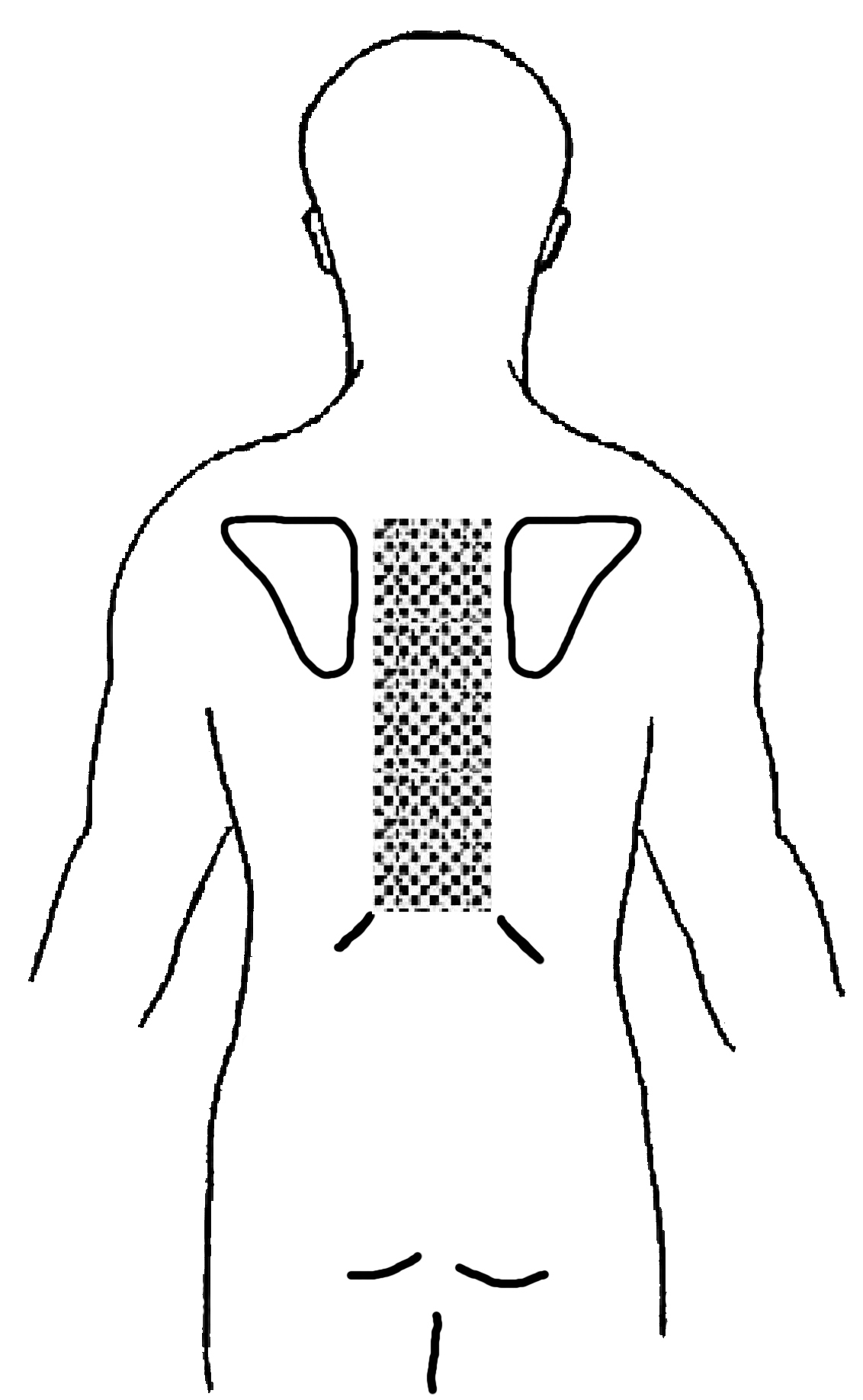  Midt på ryggen | | | | |  | |
|  | | | Person set bagfra | | | | |  | |
|  | | | | | |  | | | |
| 2a. Har du haft ondt midt på ryggen? | | | | | | 🞏 Ofte  🞏 En gang imellem  🞏 En eller to gange  🞏 Aldrig | | | |
|  | | | | | |  |  |  |  |
|  | | | | | |  |  |  |  |
|  | | | | | |  | | | |
| 2b. Har du haft ondt midt på ryggen **den sidste uge**? | | | | | | 🞏 Ja  🞏 Nej | | | |
|  | | | | | |  | | | |
| 2c. Har du ondt midt på ryggen **i dag**? | | | | | | 🞏 Ja  🞏 Nej | | | |
|  | | | | | |  |  |  |  |
|  | | | | | |  | | | |
| 2d. Sæt kryds over det ansigt, der viser hvor ondt du havde midt på ryggen, da det var værst? | | | | | | | | | |
|  | | | |  | | | | | |
| Ikke ondt |  |  | |  | | |  | | Rigtig meget ondt |
| 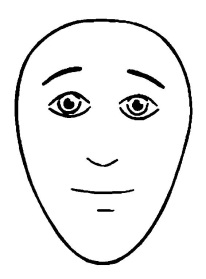 | 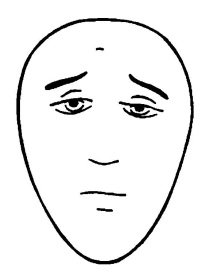 | 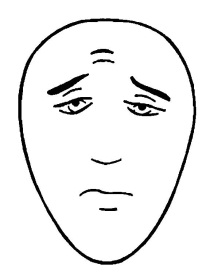 | | 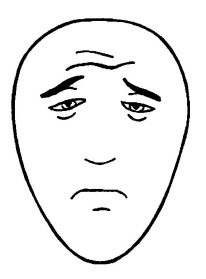 | | | 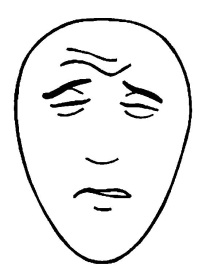 | | 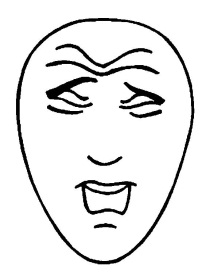 |

| **3. Lænden sidder som vist på billedet** | | | | |  | | | |
| --- | --- | --- | --- | --- | --- | --- | --- | --- |
|  | | | | |  | | | |
|  | | | 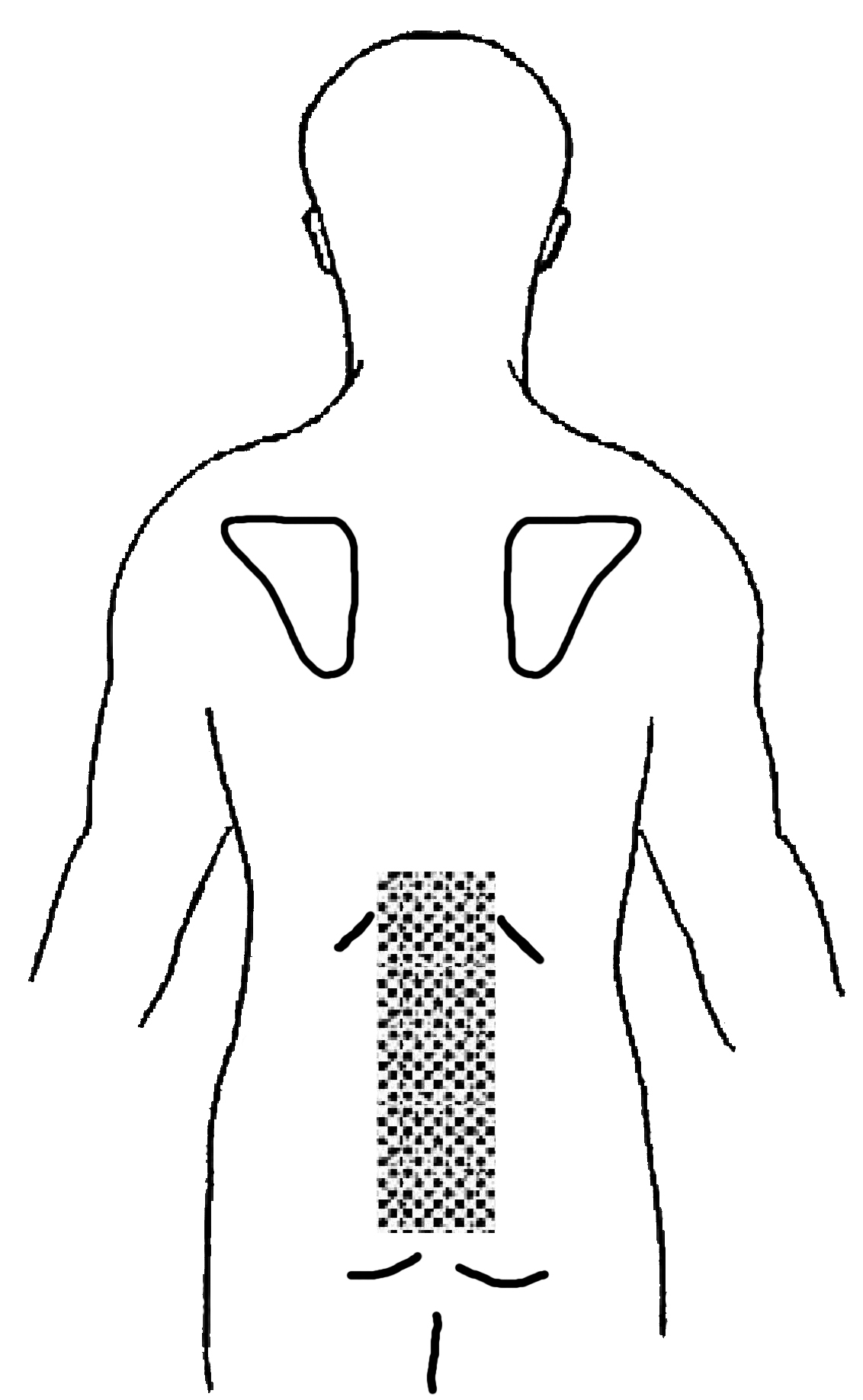  Lænden | | | |  | |
|  | | | Person set bagfra | | | |  | |
|  | | | | |  | | | |
| 3a. Har du haft ondt i lænden? | | | | | 🞏 Ofte  🞏 En gang imellem  🞏 En eller to gange  🞏 Aldrig | | | |
|  | | | | |  |  |  |  |
|  | | | | |  |  |  |  |
|  | | | | |  | | | |
| 3b. Har du haft ondt i lænden **den sidste uge**? | | | | | 🞏 Ja  🞏 Nej | | | |
|  | | | | |  |  |  |  |
|  | | | | |  | | | |
| 3c. Har du ondt i lænden **i dag**? | | | | | 🞏 Ja  🞏 Nej | | | |
|  | | | | |  |  |  |  |
|  | | | | | | | | |
| 3d. Sæt kryds over det ansigt, der viser hvor ondt du havde i lænden, da det var værst? | | | | | | | | |
|  | | | |  | | | | |
| Ikke ondt |  |  | |  | |  | | Rigtig meget ondt |
| 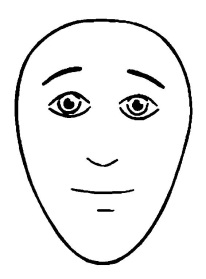 | 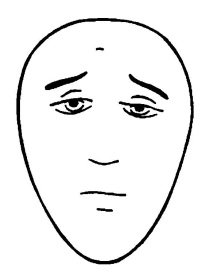 | 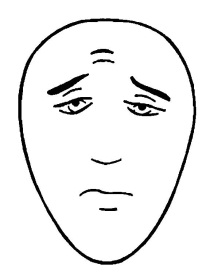 | | 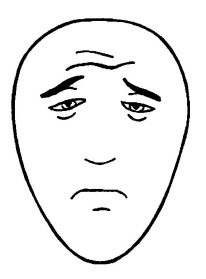 | | 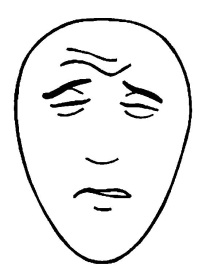 | | 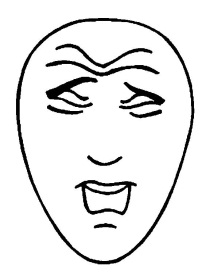 |

| **4. Skole, fritid og behandling** |  | |  |
| --- | --- | --- | --- |
|  |  | |  |
| 4a. Er du blevet hjemme fra skole på grund af ondt i ryg eller nakke? | | 🞏 Ofte  🞏 En gang imellem  🞏 En eller to gange  🞏 Aldrig | |
|  | |  |  |
|  | |  |  |
|  | |  | |
| 4b. Har ondt i ryg eller nakke forhindret dig i at dyrke idræt? | | 🞏 Ofte  🞏 En gang imellem  🞏 En eller to gange  🞏 Aldrig | |
|  | |  | |
| 4c. Har du været hos læge, kiropraktor eller fysioterapeut på grund af ondt i ryg eller nakke? | | 🞏 Ofte  🞏 En gang imellem  🞏 En eller to gange  🞏 Aldrig | |
|  | |  |  |
|  | |  |  |
| **5. Familien** | |  |  |
|  | |  |  |
| 5a Har din **far** (ham, du bor ved) nogensinde haft ondt i ryg eller nakke? | | 🞏 Ja  🞏 Nej |  |
|  | |  |  |
| 5b. Hvis han har, bliver han så hjemme fra arbejde? | | 🞏 Ofte  🞏 En gang imellem  🞏 Aldrig | |
|  | |  |  |
|  | |  |  |
| 5c Har din **mor** (hende, du bor ved) nogensinde ondt i ryg eller nakke? | | 🞏 Ja  🞏 Nej |  |
|  | |  |  |
| 5d. Hvis hun har, bliver hun så hjemme fra arbejde? | | 🞏 Ofte  🞏 En gang imellem  🞏 Aldrig | |
|  | |  |  |
